# From idea to systems solution: enhancing access to primary care in Malawi

**DOI:** 10.1186/s12913-023-09349-z

**Published:** 2023-05-25

**Authors:** L. van Niekerk, N. Fosiko, A. Likaka, C. P. Blauveldt, B. Msiska, L. Manderson

**Affiliations:** 1 London School of Hygiene and Tropical Medicine, London, UK; 2 Chembe Collaborative, Los Angeles, USA; 3 The Malawi Ministry of Health, Lilongwe, Malawi; 4 Kamuzu University of Health Sciences, Blantyre, Malawi; 5 VillageReach Malawi, Lilongwe, Malawi; 6 University of the Witwatersrand, Johannesburg, South Africa

**Keywords:** Social innovation, Primary care, Health systems, Institutionalization, Africa

## Abstract

Malawi, like many other countries, faces challenges in providing accessible, affordable, and quality health services to all people. The Malawian policy framework recognizes the value of communities and citizens, as co-creators of health and leaders of localized and innovative initiatives, such as social innovations.

Social innovations involve and include communities and citizens, as well as bring about changes in the institutions responsible for care delivery. In this article, we describe the institutionalization process of a citizen-initiated primary care social innovation, named Chipatala Cha Pa Foni, focused on extending access to health information and appropriate service-seeking behavior.

An interdisciplinary multi-method qualitative case study design was adopted, drawing on data collected from key informant interviews, observations, and documents over an 18-month period. A composite social innovation framework, informed by institutional theory and positive organizational scholarship, guided the thematic content analysis. Institutional-level changes were analyzed in five key dimensions as well as the role of actors, operating as institutional entrepreneurs, in this process.

A subset of actors matched the definition of operating as Institutional Entrepreneurs. They worked in close collaboration to bring about changes in five institutional dimensions: roles, resource flows, authority flows, social identities and meanings. We highlight the changing role of nurses; redistribution and decentralization of health information; shared decision-making, and greater integration of different technical service areas.

From this study, the social innovation brought about key institutional and socio-cultural changes in the Malawi health system. These changes supported strengthening the system’s integrity for achieving Universal Health Coverage by unlocking and cultivating dormant human-based resources. As a fully institutionalized social innovation, Chipatala Cha Pa Foni has enhanced access to primary care and especially as part of the Covid-19 response.

## Background

The Astana Declaration on Primary Health Care [1] provides a blueprint for partnerships in health with non-state actors and is built on the renewed and growing interest in innovative approaches and strong and inclusive systems responses to achieve Universal Health Coverage (UHC). To realize the goal of UHC and equitable primary health services for all, innovative localized approaches, sensitive to the realities of different social and cultural contexts, are needed [2, 3]. The WHO Framework on Integrated, People-Centered Health Services [4] emphasizes the importance of innovative primary care models, and of engaging all individuals, communities, and civil society in co-producing and being accountable for health.

However, despite communities being noted as essential for the achievement of UHC, the full engagement of communities in the design, implementation, management, and monitoring of interventions remains limited [5, 6]. Contrary to the rhetoric, programs aimed at enhancing community and public engagement are mostly top-down prescriptions, with care at best ‘co-produced’ under the guidance of an external expert, without community leadership and ownership [7]. To achieve true co-production of health, a shift is required from services being delivered to communities and citizens, to communities and citizens being recognized for their inherent strengths, given mutual responsibility, and capacitated to co-deliver care [7]. As emphasized by Odugleh-Kolve et al. [8], not only is it a fundamental responsibility for health systems to strengthen the dynamic interrelationship with patients, communities, and stakeholders, but also recognition needs to be given to their emotional, mental, and social capital which can contribute to care delivery.

Like many other African nations, Malawi faces challenges in providing accessible, affordable, and quality health services amidst a dual burden of infectious and non-communicable diseases [9]. Three key documents underpin the country’s approach to health care. First, the constitution enshrines the public provision of healthcare as a right for all Malawians [10]. Second, two health policies, the Health Sector Strategic Plan II (2017–2022) [11] and the National Community Health Strategy (2017–2022) [12], express the country’s commitment to achieving UHC, and these policies guide the implementation of health interventions within the context of the decentralized health system. Although the country has no social health insurance fund, a basic health package has been adopted [11], comprising a select number of services provided free of charge at all government health facilities. However, multiple challenges still hinder this package from being accessible and available to all Malawians. Geographic access to care is limited in rural areas although 76% of the population lives within an 8 km radius of a health facility (2016 data), and 15% of Malawians report that they are still unable to attend to their medical health needs, often due to the cost of care [13]. Women are most limited in access to care due to cost, geographic distance and associated travel time, and low levels of education [14, 15]. Facility-level issues including poor attitudes of health workers, limited availability of medicines, and long waiting times [16–18], also affect people’s use of health services. These issues are exacerbated by Malawi’s acute shortage of health professionals, which meets only 48% of national targets [19]. This equates to 1.48 health workers per 1000 population [9], far below the World Health Organization’s recommendation of 4.45 health workers per 1000 population [20].

Malawi depends on non-state actors (within both non-profit and for-profit organizations) to complement the government delivery of health services in the country, with the Christian Health Medical Association being the largest non-state provider (29%) [11]. Within this arrangement, the Malawi Ministry of Health (MoH) takes on a stewarding role, providing policy guidance and technical support. Service delivery, planning, budgeting, and monitoring are devolved to district-level structures and partners. The MoH coordinates partners through the Health Sector Working Group (HSWG), comprised of all cross-sectoral stakeholders including donors and universities. Technical Working Groups (cross-departmental and cross-sectoral thematic groups) provide technical input on various health focus areas. A key goal behind the decentralization of the Malawi health system was to achieve greater citizen participation in public affairs and policy. The National Community Health Strategy (2017–2022) [12] highlights the important role of communities as users, providers, and accountability-holders of health services. Thus, the policy framework is in place to support citizen and community-led innovative initiatives in health.

### Social innovation to extend access to primary care information

Social innovation has been presented, in both academic and policy discourse, as a promising alternate and complementary approach to achieving transformation, especially in systems plagued with complex challenges, convoluted overlaps in authority, and multiple players operating at different scales [21]. Social innovation is described as an ‘agentic, relational, situated, and multi-level process to develop, promote and implement novel solutions to social problems’ [22]. Social innovation results in institutional and systemic change [23], in five dimensions [24]: roles (creating new roles or breaking down role functions); resource flows (leveraging hidden resources or decentralizing resource distribution); authority flows (opening up the decision-making space); social identities (forming new identities and promoting interaction) and meanings (encouraging more ‘whole person’ or whole system purposes). However, for social innovation initiatives to achieve sustained systems strengthening and systems change, these initiatives need to be embedded or institutionalized across all levels of the system: at a micro level, accepted by individuals or groups; at a meso-level incorporated into organizational structures; and at a macro-level, accepted as part of the taken-for-granted structures of the health system [22, 25].

**Fig. 1:**
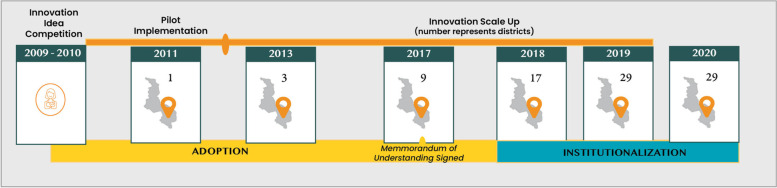
CCPF over time

In 2009, Concern Worldwide International, supported by the MoH, turned to the citizens of Malawi to crowdsource, or solicit new ideas from the public to improve maternal healthcare. At the time, Malawi had one of the highest maternal mortality ratios in Africa. The crowdsourcing competition, ‘Share an Idea, Save a Life’, led to 6047 submissions for a grand prize of $10,000. Following several stages of selection, two young Malawians with a background in information technology, Soyapi Mumba and Clement Mwazambumba, were awarded for their winning idea to provide Malawian mothers and others in their communities with health information via their mobile phones. Their intention was to bridge the gap between the community and health facilities, caused by significant distances, long waiting times, and a lack of personalized care. These citizen innovators hoped that by connecting health providers and mothers through mobile technology (voice and text messages), available accurate and timely health information would help women make better decisions about their own and their baby’s health. This was a radical idea: by 2020, mobile phone coverage had risen to 52.3 per 100 inhabitants, but in 2008, mobile phone subscription coverage was still only 10.9 per 100 inhabitants, and even less in rural areas [26]. In 2011, this idea, named ‘Chipatala Cha Pa Foni (CCPF)’ (*Health Centre by Phone)* was pilot tested and implemented by an international non-governmental organization (NGO), initially in a single district in Southern Malawi, where a central call center was established within the local government district hospital. Following a positive impact evaluation in 2013 [27], CCPF’s geographic reach was extended, and by 2016, it covered eight districts [28]. Although the initial focus was on maternal and child health, by this time, several additional health focus areas were added. In 2017 the initiative was officially adopted by the MoH, through a signed memorandum of understanding, outlining a vision to institutionalize CCPF within the public health system and serve Malawians across all 28 districts (See Fig. 1).

In this article, we present findings from studying how CCPF was institutionalized, and the institutional level changes that were required by this citizen-created initiative to embed it within the Malawi public health system.

## Methodology

A multi-method qualitative case study design was selected. This methodology is well-recognized within health policy and systems research [29] as it enables the study of ‘open systems’ where variables are not linear, and neither the phenomena nor changes over time can be controlled [30]. Social innovation is an evolving process and is highly context-bound, making a case study methodology an ideal approach [31]. This case was purposefully selected from a database on social innovations [32], because it had been scaled up and institutionalized within a low-and middle-income country.

### Data collection

A mapping exercise was undertaken to identify all relevant actors involved in the institutionalization process, and they were approached for semi-structured in-depth interviews. Interviewee categories included: implementers (creators, implementers, community collaborators); government (national and district level representatives); contributors (project partners); and other national actors who have been involved in innovation. As data collection progressed, additional participants, mainly government actors, were invited, with attention given to seeking out participants who may hold contradictory or critical opinions on the institutionalization process. A total of 68 interviews were conducted with 54 participants. Supplementary data included organizational documents (evaluations, presentations, promotional materials), monthly progress reports, and health system documents (policies). Further, observations paid attention to group processes, day-to-day management processes, actor roles, reactions, and contributions. Data collection took place in three rounds over 18 months, with 1 year of intensive engagement (July 2018 – July 2019). Interviewing and other data collection ceased when saturation was reached, and no new information was shared.

### Data analysis

Data collection commenced in the field and interim analyses were conducted between fieldwork intervals to enable iteration and triangulation. All interviews, except one, were audio-recorded and transcribed. The majority of interviews were conducted in English and selected interviews conducted in Chichewa *(the local language widely spoken)* with the help of a translator were transcribed and then translated. Field notes were typed up. All data were imported into NVivo 12, qualitative analysis software [33].

**Fig. 2:**
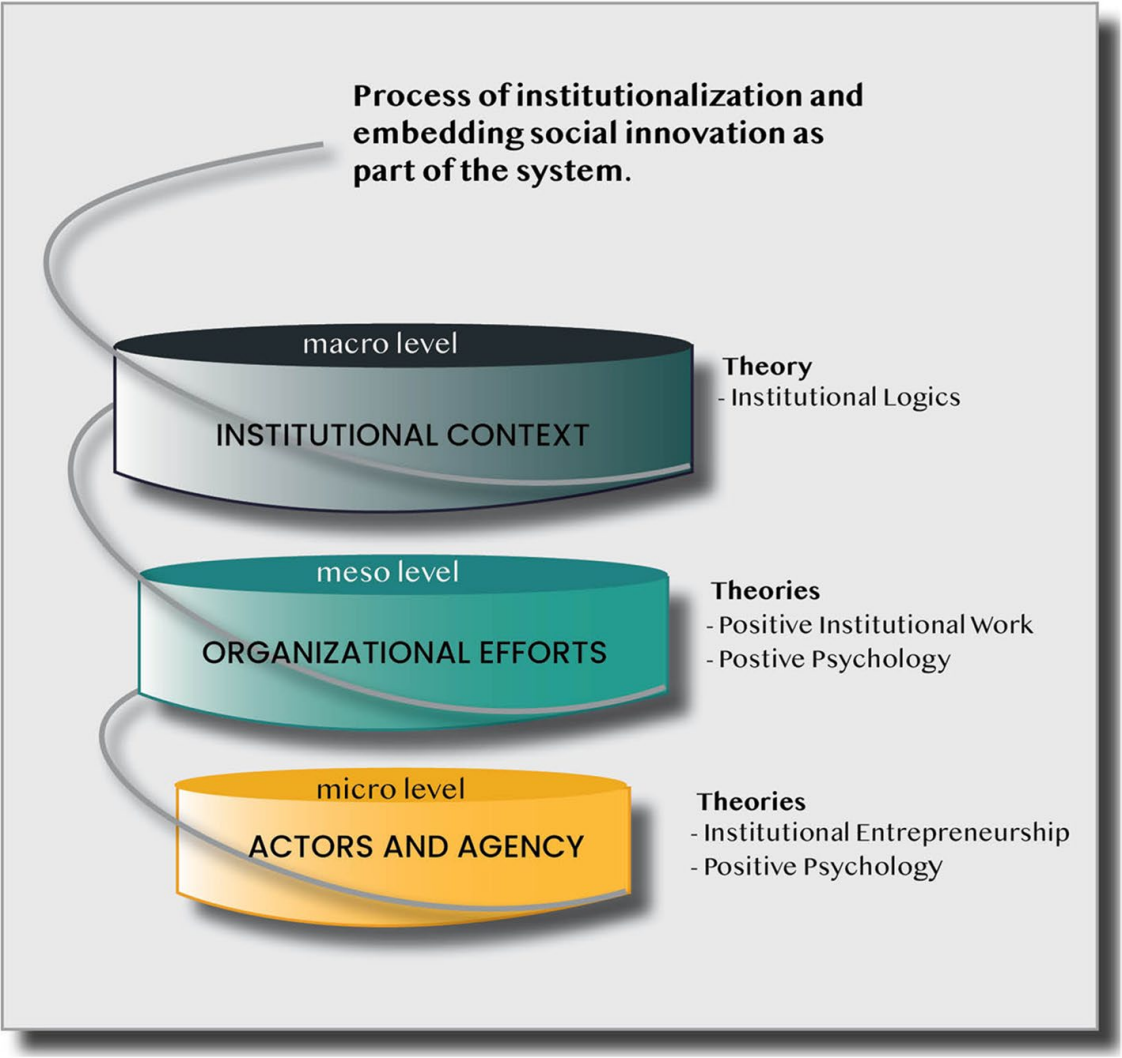
Social Innovation Framework [22]

Based on the understanding of social innovation as a process occurring across multiple levels [22], a composite framework was developed that examined the micro-individual level, the meso-organizational level, and the macro-institutional level (see Fig. 2). This framework accounting for these levels of action and change was further informed by different theories from neo-institutionalism, including institutional entrepreneurship [34] and institutional work [35, 36] (purposeful actions to bring about change), and merged with work from positive organizational scholarship [37, 38]. By combining these theories, it was possible to understand how the agentic efforts of individuals, within a resource-constrained institutional context, can lead to the creation, disruption, and maintenance in five performative institutional dimensions: roles, resource flows, authority flows, social identity, and meanings). A thematic content analysis was conducted using deductive (guided by the conceptual framework) and inductive approaches – allowing for the recognition of patterns, whereby themes (or codes) that emerge from the data subsequently become the categories for analysis [39].

### Ethics

This study received ethical clearance from the Ethics Review Board at the London School of Hygiene and Tropical Medicine and the National Council for Science and Technology in Malawi. Informed consent was obtained from all the study participants. Consent forms were made available in English *(the government and business language)* and Chichewa *(the local language widely spoken*). All methods were conducted in accordance with the approved ethical consent received for this study.

## Results

The role of actors, namely institutional entrepreneurs, their efforts, and the resulting institutional changes will be discussed below.

### Institutional entrepreneurs

**Fig. 3:**
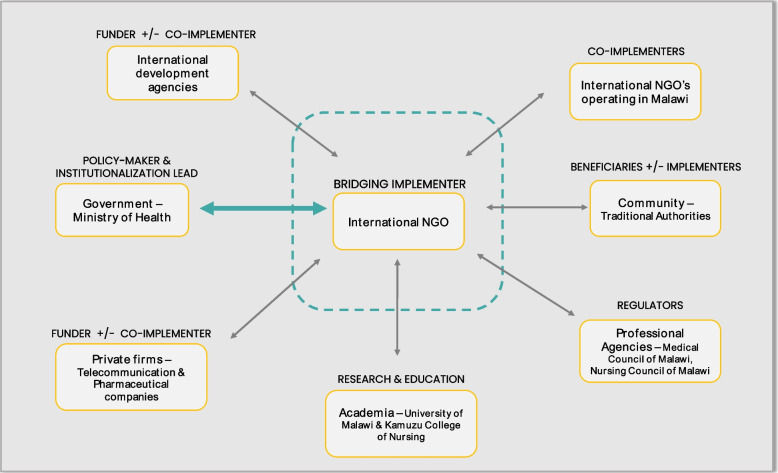
Organizations involved in the CCPF institutionalization process

Social innovations redefine boundaries and blur lines across different sectors and society [40, 41], as evidenced by CCPF. To institutionalize CCPF, organizations, and entities from across the government, the non-state sector, and the community were involved (Fig. 3). The implementing NGO served as a ‘bridging organization’ [42], a broker between the diverse interests, visions, and resources of the different constituent organizations involved. The implementing NGO was constant throughout the CCPF’s lifecycle from the pilot to its institutionalization, even though the level of involvement of the other organizations fluctuated over time.

Of the 54 actors interviewed, a subset of individual actors played a particularly important role. These were institutional entrepreneurs (IEs), defined by Battilana [43] as ‘‘actors who initiate divergent changes in the institutional context and who actively participate in the implementation’. Nine actors met this definition: three from the non-governmental sector (implementing NGO), five from the government sector (MoH), and one from the community sector (Traditional Authority). Each, in their distinct capacity, played an agentic role between 2012 and 2019, either in facilitating the adoption or in institutionalizing CCPF.

Three of the nine IEs were female and all, but one were Malawian nationals. Their backgrounds included nursing, clinical medicine, project management, and tourism. All except the community leader, held mid-level positions within their organization and they were able to make decisions or influence decisions on resources and policy adoption. Government IEs had similar career trajectories, starting at the frontlines of care delivery, holding management positions at the district level, and then being promoted to a national-level position. Compared to the NGO IEs, government IEs had a longer organizational tenure (14–26 years), which provided them with strong professional connections and alliances. Other factors which influenced their receptivity to innovation and their willingness to take on risks were attributed to their experiences gained outside of Malawi, and their prior engagement in ‘innovative’ projects (*projects deviating from the norm or standard practice*) in previous work settings.

Despite diverse career backgrounds and trajectories, IEs had a set of personal characteristics in common. All nine IEs displayed the projective dimensions of agency [44], that is, they were future-oriented or visionary. They were able to see possibilities beyond the reality of resource constraints and challenges, and this ability aligned with the positive emotion of hope. They believed in their own power to realize an innovative possibility in which they held an interest.

*“Quite often people talk about the Ministry of Health or the health system in Malawi operating in a resource-limiting environment. Though I see that sometimes, I don’t believe it is resource-limited. It just requires innovation to see how best you can do with the little you have. Some people when they see problems, they just look at them and cry. Others look at problems as opportunities, they can do something.”* (Interviewee 015 – Government)

*“There are people who would not agree and there are people who would even discourage you but when I see that there is a positive side to things, I always carry on. I am never discouraged by people that just make arguments for argument’s sake.”* (Interviewee 010 – Government).

The IEs displayed and articulated their deep passion for social change and positive impact on the lives of others around them. From observations and interviews, it appeared that this passion was not linked to specific projects but was rather something they considered as a personal characteristic. This was complemented by considerable energy and enthusiasm for life in general, and this translated to their work. They attributed their passion to childhood experiences or their Christian faith, which gave their lives a unique purpose and vision.

*“I have a passion to serve life…not serve life at a small scale but my scope is the biggest impact, that’s why I made an oath, as a medical doctor, I will work in the public service. I want to die empty…the potential in me…technically, whatever I am…God enacted in me, God who created me…. Whatever potential is in me, I will make use of it to the best of my capability. I don’t believe in what I get in my pocket but what I can contribute. That’s my passion.”* (Interviewee 038 – Government)

The IEs did not view themselves as lone heroes, but they took pride in their relational skills, collaborating with others, and building strong personal relationships. They were willing to engage in discussion, question, and debate with their colleagues. They regarded their colleagues, irrespective of their social position or status, as sources of knowledge, and believed that broad participation in processes was of great value. Each IE openly embraced the opportunity to gain new experiences and knowledge:

*“I respect each and every other person’s views. When you listen attentively there is always something you can learn, and I always view it that there is nobody who is a blank slate. We are born with some knowledge, we have some information, and you should be objective and not judgmental. I have seen those innovations indeed work, so I am positive about constructive suggestions from colleagues. Maybe that is by nature who I am.”* (Interviewee 015 – Government)

These actors initiated a series of changes in five institutional dimensions that supported the Chipatala Cha Pa Foni initiative to be embedded in the national public health system. These were: Roles, Resource Flows, Authority Flows, Social Identities and Meanings.

### Roles

For the innovation to work, a change in the institutionalized role of nurses was required. During the innovation pilot in the catchment implementation district, Health Surveillance Assistants (HSAs—community health workers) were initially appointed to attend to incoming calls. In the Malawian health system, HSAs are responsible for health promotion, disease surveillance, and basic service delivery. As part of CCPF, HSAs would provide health information and advice via mobile phones. The Nursing and Midwives Council of Malawi, initially resisted this, believing that a virtual patient-provider interaction held a higher risk than a face-to-face interaction and that the function should only be provided by a registered nurse. To keep the innovation operational, the implementing NGO appointed nurses to replace the HSAs that operated the hotline. However, even with nurses providing this service, there remained significant institutional resistance and opposition, at the time of data collection in 2019. Several interviewees felt that moving the nurse from the bedside to the telephone would compromise nurse clinical skills, knowledge, and attitudes. As one interviewee explained:

*“Yeah, I had so many concerns at first because you know, we associate nurses with only the clinical aspect, so people started having worries, what about their clinical skills, how will they aah be up to date in terms of skills.”* (Interviewee 020- Regulator)

No clear policy framework governed m-health or telemedicine initiatives in Malawi when the CCPF pilot commenced. Regulators were concerned that misinformation and incorrect decision-making would follow from the inability of the nurse to see a patient face-to-face to observe physical signs of illness, and their reliance only on dialogue. To mitigate this, it was deemed that CCPF nurses could not provide formal consultations but only provide health information and refer the caller to a health facility. A final and ongoing concern expressed by interviewees regarding the role transformation was that health facilities in Malawi already experienced acute staff shortages and that nurses involved in the innovation were lost from the frontlines. Other interviewees however regarded this as a strength of the innovation, arguing that nurses positioned in a hotline, providing information telephonically, could serve more people and do so more efficiently.

Despite these objections, two factors enabled this institutional change to happen. First, the implementing team drew on the existing legitimate function of nurses to provide health promotion and education. Second, they emphasized the limitations hindering access to health care experienced by Malawians: geographic distances, the over-congestion of health facilities, and limited (if any) patient-provider consultation time. The CCPF theory of change was that access to primary care can be improved by providing timely health information, thus ensuring that care is sought appropriately while avoiding unnecessary consultations. One IE explained how he advocated for this change:

*“I thought they (CCPF) were reaching more people, without people having to walk to the hospital because we haven’t yet reached these people. You know maybe some have 15 km, others have 10km (to walk), so I find it very interesting. At least we are assisting many people and we are trying to minimize congestion at the hospital as they will be in the queue to be told it is just minor.”* (Interviewee 014 – Government)

Although both the Nursing and Midwives Council and the Ministry of Health (MoH) approved the plan to transform the role of nurses in 2011/12, the challenge associated with this institutional change surfaced again later. As part of the institutionalization process (2017–2020), the MoH needed to incorporate the hotline nurses as government health workers, rather than having them employed directly by the implementing NGO. Within the government staff delineations, there was no staff establishment for nurses operating in a virtual advisory capacity. While this was overcome in December 2020, it was a major barrier, to institutionalization:

*“To train a health worker here is expensive. Should they be answering phones when we have a high vacancy rate in our hospitals? My thinking said ‘ah, are we ready for this?!”. It is a very good initiative, yes, but should government actually start creating positions for this when we are failing to address the challenges in our hospitals?”* (Interviewee 052 - Government)

### Resource flows

Eighty-four percent of Malawians live in rural areas and face multiple socio-economic challenges including poverty, a lack of income-generating opportunities, electricity shortages, limited mobile phone coverage, internet access, and low literacy. Deeply embedded traditional and patriarchal structures influence women’s access to health services. Before the implementation of CCPF, health information, especially in rural areas, was regarded as a privileged resource, owned by health professionals and only accessible to people visiting a health center.

With the implementation of CCPF, access to health information became more widely distributed. First, information was sent to users (via text messages) and users could call the hotline free of charge with specific health questions. Second, the hotline was later open to all Malawians, not just mothers or caregivers. One of the main Malawian private telecommunications providers established a dedicated phone number to be used free of charge by people calling the hotline and enabled text messages, customized to the user’s specified health interest, to be sent to thousands of people simultaneously.

Both interview and observation data, as well as impact evaluation data, provided evidence of the effect of the enhanced distribution of health information on community actions and agency. In the traditional authority (TA) area visited during fieldwork, the traditional leader explained that the innovation had become part of his strategy to improve the health of the 70,000 people over whom he presided, including to reduce maternal mortality. In this area, few mobile phones existed within the community, and these were mainly owned by men. To overcome this, the leader raised funds to purchase several mobile phones, and he provided these to selected women in the community called ‘secret mothers’. These women had a role to identify pregnancies in the community and provide a mobile phone to any woman who wanted to call the hotline. In addition, the health information communicated via text messages was written onto the walls of buildings all over the village. In this way, the traditional leader was able to disseminate information to raise broad awareness about antenatal care, and so address selected cultural beliefs and practices that were responsible for poor maternal outcomes. The leader simultaneously adopted bylaws to hold the community accountable when they were not adhering to these healthy practices.

*“CCPF simplified the role of traditional leaders. Instead of us moving around in the community, telling pregnant women to go and deliver in the hands of skilled personnel, to start ANC in the first trimester, telling them whatever message that we want to pass on to the women, was now simplified because most of the women could call the CCPF, then listen to the messages. It eased the workload for the personnel at the health facility, instead of attending a long queue of pregnant women who were just going to the facility just to ask for advice, now, those women could get the advice from the phones. It also gave a kind of empowerment to the pregnant women, to say, ‘when I get to the facility, what I wish to get is A,B,C,D just because they have already heard from the CCPF.”* (Interviewee 019 - Traditional Leader)

CCPF was one of the first initiatives in Malawi to leverage technology in support of primary health care provision, thus challenging the dominant community institutional logic on care provision. Communities were skeptical that phone calls to the hotline were free of charge, and that remotely located health professionals could provide women with information, such as their expected date of delivery. Community members associated this type of ‘future knowing’ with occultic fortune-telling practices and witchcraft. In response, community mobilization campaigns were strategically hosted by the implementing NGO to establish the innovation’s legitimacy:

*“The reaction was like, this is new. Globally m-Health is also very new, of course maybe now it can be 10-15 years, but we considered it new, and there were some questions from the communities for example: ‘how can a phone call be free?’. As you know Malawi phone calling tariffs are just very high. I remember facing a lot of challenges from the communities that this might be linked to these satanic things and all that. It was a bit challenging to make people fully understand what Chipatala cha pa Foni is, and we could see that people need to be oriented on what m-Health is about.”* (Interviewee 007 - Implementer)

The decentralization and re-distribution of health information, as a resource, led to greater distributed agency among community members, who when using the innovation, felt more competent to make informed decisions and improve health-seeking behavior. An independent impact evaluation [45] conducted in 2018 found that CCPF had a statistically significant impact on sexual reproductive health, HIV, maternal health, child health and nutrition indicators; that 87% of users reported following the advice provided; and that 87% appropriately sought care at a health facility when referred. Ninety-eight percent of users reported satisfaction with the service and half indicated that they would still access the service, even if they had to pay for use.

### Authority flows, social identity, and meanings

Institutional changes occurred not only due to the innovation implementation but also due to the institutionalization process. CCPF did not follow a linear implementation and institutionalization process; rather, these processes occurred concurrently, evolving according to circumstance and opportunity. An institutionalization structure of key significance was the steering committee meeting, launched in 2016. It was led by several IEs and this structure supported institutional changes in three key dimensions: authority flows (who decides what), social identities (who belongs to what), and meanings (who signifies what).

CCPF was one of the first technology-enabled cross-departmental, cross-boundary initiatives institutionalized in the country. The IEs driving the process were aware from the beginning that financial resources and authority flows are distributed across various health system actors, and thus they considered it important to move away from a top-down command-control approach of leadership and decision-making. They adopted a more collaborative approach with distributed decision-making. As one partner of the project explained:

*“Definitely the stakeholder collaboration is key. I think that there is a steering committee, and the NGO has been the glue that has kept all the stakeholders together, making sure the communication lines are open and make sure everyone is on the same page. I think that has helped to bring it (CCPF) to where it is now.”* (Interviewee 026- Partner)

The steering committee meeting structure unlocked the creativity needed to support a process of ‘innovating to institutionalize’. Observational and interview data showed that this second process of innovation involved less institutional disruption than occurred during the pilot. Rather, the institutionalization process focused on developing creative embedded strategies to support the introduction of CCPF within existing institutional boundaries and health system constraints. Within the public sector, system integrity had to be maintained and could not undergo a total redesign, as one government participant explained:

*“I think it is both: the innovation cannot be rigid, it has to be flexible, for example now, we are taking up Chipatala Cha Pa Foni as part of the Ministry of Health or the health systems in Malawi. There are policies, so the innovation cannot be exactly the way it was, it (CCPF) has to be modified a bit to suit the system but at the same time the system has to respond to welcome this, it (the system) has to adjust in some areas to make sure that this is set. So, it’s both ways.*” (Interviewee 015 – Government)

Specific practices were important in ‘facilitating changes in authority flows, social identities, and meanings. These practices, discussed further below, included: hosting a space that allowed for creative engagement [46, 47]; vertical and horizontal inclusivity [48] that gave all actors an equal opportunity for contribution; and shared leadership which allowed new IEs to play a role [49–51]. The IE who chaired this meeting was key to facilitating a shared space for discussion, collaboration, creativity, and relational engagement among various partners. Vertical and horizontal inclusivity was achieved by including all actors: representatives from 10 different technical departments within the MoH, from district to national level; different levels of project implementers (from the director to the hotline nurses); partners representing different sectors – private companies, bilateral development agencies, NGOs, academia, and professional associations; and beneficiaries. Any given meeting would be attended by 20 – 40 people. Meeting structures such as these were not unique in the Malawian health system, as multiple multisectoral technical working groups gather at regular intervals around topic areas. However, from observation data, the vertical and horizontal inclusivity across levels of hierarchy and power was unique:

*“But I learned from one of our steering committee meetings some people in the village have been using CCPF. It was very amazing to see that this person with his phone. He was able to give health messages to almost an entire village and the entire village was able to ask about anything that is related to health, by using his cell phone.”* (Interviewee 018 – Government)

The leadership style of the steering committee meeting chair did not project downward influence on followers (vertical leadership), but rather demonstrated shared leadership [49–51], allowing for two emerging IEs from the MoH to gain confidence and play a leading role in institutionalization.

All steering committee meeting participants were given transparent access to information related to the innovation, such as actions implemented, the latest monitoring data, and challenges faced. Steering committee meeting participants at any level could propose ideas, suggestions, and solutions for how the innovation might continue to develop and adapt to become institutionalized. All were part of the decision-making process on the course of action. Steering committee meeting participants also all contributed resources within their realm of influence – personal or professional networks, financial or in-kind resources, and opportunities for advocacy and promotion.

*“The collaboration that is there between our partners, [the NGO] and Ministry, has strengthened because we can discuss together. If there are issues, we sit and discuss them among ourselves. Like I said, even the steering committee has incorporated various departments, so it is like everybody is aware. They are left out, and then they will be surprised ‘I don’t know that this is happening’ so there is such transparency and the involvement of different people.”* (Interviewee 010 - Government)

These practices supported the creation of a shared social identity and emotional solidarity among steering committee meeting participants, often talking about CCPF as ‘this is ours’. This new identity created by the innovation overcame the diversity and distinction between individual institutional identities and assisted with addressing implementation and institutionalization challenges.

*“It is through the meetings we had; I think we had the first meeting. I don’t know where I was first involved but I found it already there. My colleague was the first one to be involved in one of the meetings and I also have been to attend one of them. Ja. It was quite good. And after that, it is like we are moving together, with the clinical department. Ja, so we share ideas and when there is a meeting we go and then see how we are moving forward.”* (Interviewee 009 – Government)

The generation of positive emotions also supported the creation of a shared social identity. These positive emotions, such as hope and pride, were first identified within IEs, and they played a role in catalyzing and amplifying these emotions in the broader group.

By March 2019, during the second data collection visit, it was clear that the MoH faced two major challenges in fully institutionalizing the initiative by the intended date of 30 June 2019. The first was difficulties in adding 29 additional nurses into the government establishment (i.e., having the necessary funding to create new positions), especially with the resource constraints of the Malawian government and budget cuts in the 2019 financial year. Second, the ongoing operational running costs of the innovation exceeded the funding available to the managing department”. During steering committee meetings, participants critically engaged to identify obstacles and express their own doubts. They were also able to reflect upon decisions that may have had negative and unintended consequences on the process. Yet, as illustrated below, all institutional entrepreneurs and the steering committee participants were resolute that institutionalization would eventually be possible:

*“There are two scenarios I am seeing because, I believe come July [the institutionalization deadline], we will not have a human resource ready. Come July we will not have financial resources. Come July we will not have good monitoring and evaluation. So, if handover indeed happens in July, then it will crash, and they will start picking it up from the ground. But for me, I think it’s a wonderful innovation and there is a lot of things that can be done. I just hope the government will give it all the support that is needed, I know some people are very interested and have ideas to take it to another level. I think we should expect that we will have challenges, but the good thing is to believe that if we fall, we will try again.”* (Interviewee Government 045– District)

This future, possibility-oriented and visionary view despite significant challenges conferred the positive emotion of hope. Unlike other positive emotions, hope does not arise in settings where circumstances are perceived as safe. Rather, it comes alive in circumstances when people might fear the worst but yearn for better [38]:

*“To me it is a mark for Malawi. It is something people said couldn’t work, it was a waste of time, a waste of resources but there it has worked. So, to me, it is possible, once you have enough information, recommendations, or findings that this can make a difference in society. Believe in yourself, believe in what you’re doing.”* (Interviewee 051 - Government)

*“I think when we get to have a steering committee meeting and he (steering committee meeting chair) says for instance: ‘[name], I am not worried about money. I know this thing must run and it will run. Money will be made available, that is the least of my concerns’. At one point I was concerned that the budget that was passed represents, maybe 10% or somewhat of what it takes to run CCPF. But if he is quite confident to say that is not his problem, then why should it give me a headache. So, when you have those discussions with Dr, you go into the room nervous but as you are leaving the room, you go, I think I need to rethink it all and get to the level of confidence that he has.”* (Interviewee 002- Implementer)

The broader group of steering committee meeting participants rotated, and government representatives were often assigned to attend. Thus, it is unlikely that this group attracted high-hope individuals, such as IEs, by self-selection. Rather, hope was likely generated in the context of the steering committee meetings. In the broader group, a sense of hope and hopefulness was observed, as exemplified by this frontline nurse who attended the meeting and whose job security depended on successful institutionalization:

*“Yes! We have invested a lot and it will be bad to just give up now. We still have hope that it, though it will not go as we wanted, as we anticipated, we still have hope that it will go through”* (Interviewee 003 - Implementer).

Pride was also widespread among steering committee meeting participants at the group level. Members viewed the initiative as a way to positively impact their country and they felt that, as Malawians, they could then take credit for implementing an initiative that few other African countries have done. All actors engaged in the institutionalization of CCPF did so on a voluntary, non-renumerated basis, contributing over and above their daily work tasks and functions. Pride is an intangible resource, in the perseverance of ongoing efforts by actors [52].

*“To contribute something towards the development of my country, that is the most important thing, because when you see people that are using something to which you contributed, you feel good. You know, people are healthy because of my contribution. My country is developing because of what I did at the meeting. I would be very happy if the initiative is continuing because a lot of people can benefit from the service.”* (Interviewee 051 – Government District)

*“I think seeing this project come from just being on paper and then we get feedback, ‘oh my gosh! we got 20-something thousand phone calls from this.’ And then the testimonies, meeting the people that this initiative has impacted is just so heart-warming and so fulfilling for me. I mean, come on! We can see the impact from this woman who was able to be assisted by just a phone call. So yeah, it is really awesome!”* (Interviewee 026- Private partner)

The final institutional change facilitated by CCPF was in terms of meanings—the explanations for the way things are [53]. Government health institutions are organized according to functional roles and disciplines e.g., a nursing department oversees functions relating to nurses. Yet, findings suggest that CCPF cut across siloed operational, organizational, disciplinary, and sectoral boundaries. By 2018, it had grown to become a comprehensive technology-enabled service delivered by nurses who provided information on all health topics, 24-h a day, 7-days a week, for all people (children, adolescents, working adults, elderly). The institutionalization of CCPF did not come without tension in meanings. This occurred in line with that which is expected from social innovation — approaches which are more whole-person and holistically oriented, contrasted with the traditional siloed (vertical) healthcare approach, organized according to functional areas.

As noted, CCPF required the involvement of multiple departments within the MoH, to ensure that the service was implemented according to existing health policy standards and guidelines. A situation that caused significant tension in meanings, related to which department should organizationally ‘house’ this initiative such that leadership could be provided. CCPF was moved from one department to another within the ministry, but frequently respondents questioned whether it would not be better placed in a technical department dealing with health promotion. Several steering committee meeting participants proposed options such as creating a dedicated unit within the government structure. This was not resolved at the time of data collection completion, but as observed, it did stimulate government actors to reflect on an appropriate structure to support this social innovation:

*“To me, I think, integration is key because in a health facility, there are different players that are interested in their services that are being delivered. So, bringing those service providers together as what we have done, people from different departments coming together as one team to discuss on how we run this, it’s something that I feel is an innovation on its own.”* (Interviewee 051B – Government)

*“So, from the way I understand that, at first, I think there were discussions around where CCPF should be positioned, should it be positioned at the district level, in this case, the Lilongwe DHO. Or should it be attached at the hospital level, at the Kamuzu Central Hospital? Or, if it should fall within the Clinical Services Directorate. So, I think members who were present at the transition meetings suggested that it shouldn’t be tied to any of these three, it should be an individual entity of its own. It is not going to be a department, but it is rather going to be like a unit.”* (Interviewee 002 – Implementer).

## Discussion

We have highlighted three contributions of social innovation initiatives to health systems: supporting greater access to primary care for all people; strengthening health system integrity; and nurturing human-based resources in health systems. We discuss these further below.

The Chipatala Cha Pa Foni (CCPF) was a citizen-led idea that, through the support of an implementing NGO, improved access to health information for all Malawians. On completion of data collection in July 2019, the social innovation had not yet succeeded to be integrated into the Malawi Ministry of Health (MoH), although it was scaled to all 28 districts in Malawi. Full institutionalization was achieved in December 2020, and henceforth, the social innovation is fully owned and managed by the Malawi MoH. This primary care social innovation proved especially valuable during the Covid-19 pandemic when, now part of a newly created Emergency Medicine Unit, it became a key component of Malawi’s pandemic response, at a time when access to accurate health information was critical. The government adapted and utilized CCPF’s capabilities to triage people with Covid-19 symptoms, do contact tracing, patient monitoring, mapping patient journeys, educate and dispel Covid-19 myths and create referral linkages between the health system and social services.

CCPF resulted in changes in five key institutional dimensions. These changes make it possible to classify this initiative as social innovation. The institutional changes did not seek to disrupt the health system; rather the initiative supported strengthening the system integrity. It did so in numerous ways: bringing new health system leaders to the forefront (institutional entrepreneurs); facilitating greater collaboration between government, citizens, and non-state actors; and building capacity for collective creativity. Health systems could thus approach social innovation not as a risk but as a way to strengthen systems integrity, as all the socio-cultural capacities generated have transferrable benefits by application in other existing or future health system initiatives. The socio-cultural systems change which occurred by actors collaborating on a tangible social innovation initiative can provide a subtle and arguably subversive way by which human and other dimensions of health systems can be nurtured, beyond only enhancing programmatic care delivery and health outcomes. This reduced concern by decision-makers that social innovation institutionalization would hinder and distract from ongoing agendas and efforts to build stronger health systems. Rather it provided direction to social innovation’s complementary contribution in institutional strengthening and supporting the achievement of people-centered health systems.

Social innovation also cultivated human-based resources in health systems such as positive emotions. Institutional entrepreneurs matched up with Snyder’s understanding of hope as “the sum of the mental willpower and waypower that you have for your goals” [54]. As above, all had mental willpower—the perception that they could initiate actions to achieve the goal—and waypower—the ability to find alternate routes towards achieving their goal if obstacles presented. These qualities identified in the data also differentiate hope from optimism. Optimistic individuals hold fast to the belief of a positive outcome but lack critical thinking about how to arrive there. However, the CCPF actors, especially the nine institutional entrepreneurs, can better be described as high-hope individuals [54, 55], who could influence a larger group, as they unlocked and raised hope levels dormant within a larger group. Raising collective hope levels (future-oriented agency) subsequently became a catalyst towards further action (habitual and practical evaluative agency) and relentless determination, as required to institutionalize a social innovation [44].

The nurturing ‘ground’ for hope came from shared interactions and experiences created by the steering committee meetings. In pressured health system contexts, many meetings may occur, but the components of vertical and horizontal inclusivity, transparency, shared leadership, and creative participation gave way to relational bonds strengthened between members of the steering committee. Through high-hope individuals, in relationship with other members, hope was injected into the larger group. This sustained the ongoing efforts to institutionalize the social innovation, despite the challenges. This created a more positive experience for Malawi nationals, in contrast to their usual experience when innovative initiatives are implemented from outside the system and by non-nationals. The social innovation process, if applied well and with sensitivity, could be an alternate way to overcome some of the past and present colonizing implementation practices.

## Conclusion

Social innovations are initiatives that bring about changes within the institutional and social-cultural dimensions of health systems. In terms of institutional change, the Chipatala Cha Pa Foni, as a social innovation led to a change in the roles of nurses and a change in the flow of health information. In terms of social-cultural change, the role of actors, operating as institutional entrepreneurs, within the social innovation institutionalization process, played a key role. These actors strategically drove the social-cultural change through engaging in shared leadership, creative collaboration, and drawing on their inherent human-based resources such as hope. Institutionalized citizen-led social innovation receiving strong government support can be a practical way to improve primary care access for the achievement of Universal Health Coverage.

## Data Availability

The datasets generated and analyzed during the current study are not publicly available due to the confidentiality risk posed to participants. The datasets generated and analyzed during the current study are not publicly available due to the confidentiality risk posed to participants. The datasets used and/or analysed during the current study available from the corresponding author on reasonable request.
