# Correction to: From idea to systems solution: enhancing access to primary care in Malawi

**DOI:** 10.1186/s12913-023-09798-6

**Published:** 2023-07-12

**Authors:** L. van Niekerk, N. Fosiko, A. Likaka, C. P. Blauvelt, B. Msiska, L. Manderson

**Affiliations:** 1 London School of Hygiene and Tropical Medicine, London, UK; 2 Chembe Collaborative, Los Angeles, USA; 3 The Malawi Ministry of Health, Lilongwe, Malawi; 4 Kamuzu University of Health Sciences, Blantyre, Malawi; 5 VillageReach Malawi, Lilongwe, Malawi; 6 University of the Witwatersrand, Johannesburg, South Africa

**Correction to: *BMC Health Services Research*** **(2023) 23:547.**

10.1186/s12913-023-09349-z

Following publication of the original article [1], the authors identified an error in the author name of the 4th author.

The incorrect author name is: C. P. Blauveldt.

The correct author name is: C. P. Blauvelt.

The author group has been updated above and the original article [1] has been corrected.
